# Tonic Modulation of Spinal Hyperexcitability by the Endocannabinoid Receptor System in a Rat Model of Osteoarthritis Pain

**DOI:** 10.1002/art.27698

**Published:** 2010-12

**Authors:** Devi Rani Sagar, Lydia E Staniaszek, Bright N Okine, Stephen Woodhams, Leonie M Norris, Richard G Pearson, Michael J Garle, Stephen P H Alexander, Andrew J Bennett, David A Barrett, David A Kendall, Brigitte E Scammell, Victoria Chapman

**Affiliations:** 1 University of Nottingham and Queen's Medical CentreNottingham, UK; 2 University of NottinghamNottingham, UK

## Abstract

**Objective:**

To investigate the impact of an experimental model of osteoarthritis (OA) on spinal nociceptive processing and the role of the inhibitory endocannabinoid system in regulating sensory processing at the spinal level.

**Methods:**

Experimental OA was induced in rats by intraarticular injection of sodium mono-iodoacetate (MIA), and the development of pain behavior was assessed. Extracellular single-unit recordings of wide dynamic range (WDR) neurons in the dorsal horn were obtained in MIA-treated rats and saline-treated rats. The levels of endocannabinoids and the protein and messenger RNA levels of the main synthetic enzymes for the endocannabinoids (*N*-acyl phosphatidylethanolamine phospholipase D [NAPE-PLD] and diacylglycerol lipase α [DAGLα]) in the spinal cord were measured.

**Results:**

Low-weight (10 gm) mechanically evoked responses of WDR neurons were significantly (*P* < 0.05) facilitated 28 days after MIA injection compared with the responses in saline-treated rats, and spinal cord levels of anandamide and 2-arachidonoyl glycerol (2-AG) were increased in MIA-treated rats. Protein levels of NAPE-PLD and DAGLα, which synthesize anandamide and 2-AG, respectively, were elevated in the spinal cords of MIA-treated rats. The functional role of endocannabinoids in the spinal cords of MIA-treated rats was increased via activation of cannabinoid 1 (CB_1_) and CB_2_ receptors, and blockade of the catabolism of anandamide had significantly greater inhibitory effects in MIA-treated rats compared with control rats.

**Conclusion:**

Our findings provide new evidence for altered spinal nociceptive processing indicative of central sensitization and for adaptive changes in the spinal cord endocannabinoid system in an experimental model of OA. The novel control of spinal cord neuronal responses by spinal cord CB_2_ receptors suggests that this receptor system may be an important target for the modulation of pain in OA.

Joint disease such as osteoarthritis (OA) is associated with chronic pain (1). The mechanisms underlying OA pain are widely acknowledged to be complex. Recent evidence suggests that central sensitization may contribute to pain in patients with OA (2). Studies using models of knee joint pathology are essential for further understanding of the mechanisms leading to chronic pain in patients with OA. Intraarticular injection of the glycolysis inhibitor sodium mono-iodoacetate (MIA) produces cartilage and subchondral bone pathology consistent with that seen in human OA joints (3,4) and a pronounced decrease in weight-bearing on the injured hind limb (5–7), indicative of hyperalgesia. Furthermore, consistent with the notion of central sensitization, tactile allodynia is exhibited by the hind paw ipsilateral to the joint pathology (6,8). These behavioral data suggest that there are changes in spinal cord processing of afferent input in this model of OA pain. This is consistent with the observation that sensitization of joint nociceptors increases the peripheral receptive field size of neurons innervating the knee joint, paw, and ankle in rats (9).

The endocannabinoids have well-described roles in the modulation of nociceptive processing (10). Indeed, increased activity of the nociceptive pathways is associated with increased levels of the endocannabinoids, particularly anandamide, in the dorsal root ganglia (11) and spinal cord (12,13) in models of neuropathic pain. Importantly, there is evidence for increased peripheral endocannabinoid-mediated control of the mechanosensitivity of afferent nerve fibers in the MIA-induced OA model (14). Elevated levels of endocannabinoids in models of chronic pain are likely to counteract the increased neuronal activity driven by afferent input and, therefore, may provide inhibitory modulation of the mechanisms driving central sensitization. Maintenance of these elevated levels of endocannabinoids by the manipulation of catabolic enzymes is effective for decreasing pain behavior in other models of pain (10).

The aim of this study was to investigate the relationship between joint pathology, tactile allodynia, and spinal neuron responses in the MIA model of OA pain and to determine whether spinal cord endocannabinoids tonically control the noxious versus innocuous responses of neurons. We report, for the first time, that this experimental model of OA is associated with increased spinal cord levels of the inhibitory endocannabinoids anandamide and 2-arachidonoyl glycerol (2-AG) and increased protein levels of the major enzymes responsible for their synthesis, *N*-acyl phosphatidylethanolamine phospholipase D (NAPE-PLD) and diacylglycerol lipase α (DAGLα). We demonstrate endocannabinoid-mediated inhibitory control of spinal cord neuronal responses via the activation of spinal cannabinoid 1 (CB_1_) receptors and a novel functional role of spinal CB_2_ receptors. Blockade of endocannabinoid catabolism by the fatty acid amide hydrolase (FAAH) inhibitor URB597 inhibited low weight–evoked neuronal responses in the rat model of OA, but not in control rats, further supporting the notion of a novel functional role of spinal cord endocannabinoids in this model of OA.

## MATERIALS AND METHODS

### Animals

Studies were carried out in accordance with the UK Home Office Animals (Scientific Procedures) Act (1986) and followed the guidelines of the International Association for the Study of Pain. A total of 188 male Sprague-Dawley rats (obtained from Charles River UK) weighing 160–190 gm were used. Anesthetized rats received a single intraarticular injection of MIA (0.3 mg/50 μl, 1 mg/50 μl, or 3 mg/50 μl, based on previous studies) in saline through the infrapatellar ligament of the left knee.

### Behavioral testing

The experimenter was blinded to treatments. Baseline measurements were obtained immediately prior to intraarticular injection (postoperative day 0) and then from postoperative day 2 to day 28. The effects of intraarticular injection of MIA or saline on weight distribution through the left (ipsilateral) and right (contralateral) knee were assessed using a Linton Incapacitance Tester (Linton Instrumentation), as previously described (15). The development of hind paw tactile allodynia was assessed using von Frey monofilaments (Semmes-Weinstein monofilaments [bending forces of 1 gm, 1.4 gm, 2 gm, 4 gm, 6 gm, 8 gm, 10 gm, and 15 gm]), as previously described (16). Von Frey monofilaments were applied, in ascending order of bending force, to the plantar surface of both hind paws. The lowest weight of monofilament that elicited a withdrawal reflex was recorded as the paw withdrawal threshold.

### In vivo electrophysiology

The methods used were similar to those previously described by Chapman et al (17). Rats were anesthetized with isoflurane (3% for induction, 2% during surgery, 1–1.5% for maintenance in 66% N_2_O, 33% O_2_), placed in a stereotaxic frame, and a laminectomy was performed to expose segments L4–L5 of the spinal cord. Core body temperature was maintained at 36.5–37.5°C. Extracellular single-unit recordings of deep wide dynamic range (WDR) dorsal horn neurons were obtained with glass-coated tungsten microelectrodes. Action potentials were digitized and analyzed using a CED micro1401 interface and Spike 2 software (Cambridge Electronic Design). Neurons that responded to brush and pinch stimuli were identified, and their depths from the spinal cord surface were recorded (mean ± SEM depth of neurons 806 ± 16 μm). The responses of WDR neurons to mechanical punctate stimuli applied to the peripheral receptive field on the hind paw were characterized. Von Frey monofilaments (Semmes-Weinstein monofilaments, calibration codes 5.07, 5.18, 5.46 corresponding to bending forces of 10 gm, 15 gm, and 26 gm, respectively) were applied to the plantar surface of the receptive field for 10 seconds, as previously described (16); the number of evoked action potentials was recorded, and the frequency of firing over this period of time was calculated. Data are expressed as the mean frequency of firing or as the percent of pre–drug control responses.

### Spinal drug administration

One WDR neuron per rat was used for the pharmacologic studies. The effects of direct spinal administration of the CB_1_ receptor antagonist AM251 (0.1–10 μg/50 μl; n = 6 neurons in 6 MIA-treated rats and n = 6 neurons in 6 saline-treated rats) or vehicle (3% Tween 80 in saline; n = 6 neurons in 6 MIA-treated rats and n = 6 neurons in 6 saline-treated rats) on mechanically evoked responses (as described above) of WDR neurons were studied. In a separate group of rats, the effects of spinal administration of the CB_2_ receptor antagonist SR144528 (0.001–0.1 μg/50 μl) on mechanically evoked responses of WDR neurons were studied (n = 6 neurons in 6 MIA-treated rats and n = 6 neurons in 6 saline-treated rats). Note that the same vehicle was used for AM251 and SR144528. The effects of inhibiting catabolism of the endocannabinoids by FAAH with URB597 (10–50 μg/50 μl) (18) on mechanically evoked responses of WDR neurons were studied (n = 6 neurons in 6 MIA-treated rats and n = 6 neurons in 6 saline-treated rats). The same vehicle as that described above was also used for URB597.

### Measurement of endocannabinoids

In separate groups of MIA- and saline-treated rats, spinal cord levels of endocannabinoids on days 14 and 28 postinjection (n = 10 rats per group) were measured using an established method (19), with minor modifications as indicated below. The ipsilateral and contralateral lumbar spinal cord was dissected and stored at −80°C; samples were minced and added to ice-cold acetonitrile containing internal standards (0.42 nmoles d8-anandamide, 1.5 nmoles d8–2-AG). Simultaneous measurement of endocannabinoids and related compounds was then performed using liquid chromatography–tandem mass spectrometry. Analysis was carried out on an Agilent 1100 system coupled to a Quattro Ultima triple quadrupole mass spectrometer (Waters) in electrospray-positive mode. Analytes were separated chromatographically on a Waters Symmetry C18 column (internal diameter 100 × 2.1 mm, particle size 3.5 μm), with a mobile phase flow rate of 0.3 ml/minute. Multiple-reaction monitoring of individual compounds, using specific precursor and product mass/charge ratios, allowed simultaneous measurement of anandamide, 2-AG, palmitoylethanolamide (PEA), and oleolylethanolamide (OEA).

### Enzyme assays

In an additional cohort of MIA-treated rats and saline-treated rats, the ipsilateral and contralateral lumbar spinal cord was dissected on days 14 and 28 postinjection and stored at −80°C (n = 6 rats per group). FAAH and monoacylglycerol lipase (MAGL) activities were assessed in postnuclear supernatant samples (1,000 gm) in the presence of 2 μ*M* ^3^H-labeled anandamide and 100 μ*M* ^3^H-labeled 2-oleoylglycerol (both from American Radiolabeled Chemicals), respectively, at pH 7.4 (20). Specific FAAH and MAGL activities were defined by the presence of 10 μ*M* URB597 and 1 μ*M* methylarachidonoylfluorophosphonate (Cayman Europe), respectively.

### Antibodies

Mouse primary monoclonal antibodies to β-actin (1:5,000) were obtained from Sigma. Rabbit primary polyclonal antibodies to FAAH (1:200), MAGL (1:200), and NAPE-PLD (1:200) were obtained from Cayman, and those to DAGLα (1:200) were obtained from Frontier Bioscience. The secondary antibodies used were IRDye 680–conjugated goat polyclonal anti-mouse IgG (1:10,000) and IRDye 800–conjugated goat polyclonal anti-rabbit IgG (1:10,000) (Li-Cor).

### Western blotting

The ipsilateral lumbar spinal cord was homogenized in 1 ml of radioimmunoprecipitation assay lysis buffer. The supernatant was separated from the pellet and assayed for total protein concentration, using the Pierce BCA Protein Assay Kit. Fifty micrograms of protein was separated on a 12% sodium dodecyl sulfate–polyacrylamide gel and transferred onto a Hybond ECL membrane (GE Healthcare Biosciences) The membrane was incubated overnight at 4°C with the appropriate primary antibody. Blots were scanned for densitometric analysis using the Li-Cor Odyssey Infrared Imaging System.

### RNA extraction and complementary DNA (cDNA) synthesis

The frozen ipsilateral spinal cord was homogenized in 2 ml of ice-cold TRI Reagent (Sigma-Aldrich), and RNA was purified according to the manufacturer's instructions. For cDNA synthesis, 250 ng of total RNA was reverse transcribed using SuperScript III reverse transcriptase (Invitrogen) in a total reaction volume of 20 μl. Reactions were incubated for 10 minutes at 25°C and 1 hour at 50°C, and the reaction was terminated by incubation at 70°C for 15 minutes.

### TaqMan quantitative real-time polymerase chain reaction (PCR)

Gene expression was quantified using the relative standard curve method based on TaqMan quantitative real-time PCR, as previously described (21). Primers and probes (FAM, TAMRA modified) were designed using Primer Express version 3 software (Applied Biosystems) or were obtained from previously published work and were synthesized at MWG Biotech. The primers and probes for MAGL were as follows: forward primer TGCCATCTCCATCCTAGCAG, reverse primer CAAGGATATGTTTGGCAGGAM, probe ATCCGGAATCTGCATCGACTTTGA. The FAAH probe and primers were those described by Bortolato et al (22), as follows: forward primer CTCAAGGAATGCTTCAGC, reverse primer GCCCTCATTCAGGCTCAAG, probe ACAAGGGCCACGACTCCACACTGG. The primers and probe for NAPE-PLD were as follows: forward primer CAAGCTCCTCTTTGGAACC, reverse primer CTGGAGGAGGACGTAACCAA, probe TATCCCAAACGTGCTCAGATGGCT. For DAGLα, the primers and probe were as follows: forward primer ACCTGCGGCATCGGTTAG, reverse primer CTTTGTCCGGTGCAACAG, probe CAGCTGGTCCCGCCGTCTAAAAGTG.

### Joint histology

Joints were fixed in 10% formal saline and decalcified in an aqueous EDTA solution (14% in distilled water, pH 7.0, 20°C). Samples were paraffin-embedded, and 5–8-μm sections of the central portion of the knee joint, in the coronal plane, were stained with Safranin O–fast green to show matrix proteoglycan and overall joint morphology. The medial and lateral knee compartment tibial plateau cartilage, tibial subchondral bone, and joint synovium were scored.

Articular cartilage was scored using a modified Osteoarthritis Research Society International cartilage histopathology assessment system based on a scale of 0–6, where 0 = normal, 1 = surface intact, 2 = surface discontinuity, 3 = vertical fissures, 4 = erosion, 5 = denudation, and 6 = deformation, combined with a stage score (scale of 0–4) indicating the surface extent of joint involvement (0 = no activity, 1 = <10%, 2 = 10–25%, 3 = >25–50%, and 4 = >50%) (23). Subchondral bone was assessed (4) as follows: 0 = no subchondral lesions with cellular infiltration; 1 = 1–2 subchondral lesions, <5% of the tibial plateau; 2 = 2–3 subchondral lesions, <15% of the tibial plateau; 3 = 4–5 subchondral lesions, <25% of the tibial plateau; and 4 = ≥5 subchondral lesions, >25% of the tibial plateau. Synovial hyperplasia was assessed as the presence of hypercellularity (24). A mean score for the medial and lateral joint compartments was obtained (0 = lining cell layer 1–2 cells thick, 1 = lining layer 3–5 cells thick, 2 = lining layer 6–8 cells thick and/or mild increase in cellularity, 3 = lining cell layer >9 cells thick and/or severe increase in cellularity).

### Statistical analysis

Changes in weight distribution and the development of mechanical allodynia in MIA-treated versus saline-treated rats were analyzed using two-way analysis of variance with Bonferroni's post hoc test. Comparisons of mechanically evoked responses of WDR neurons in MIA-treated and saline-treated rats, endocannabinoid levels in the spinal cords of MIA-treated and saline-treated rats, and the effects of drug interventions versus treatment with vehicle on neuronal responses were performed using a nonparametric Mann-Whitney test. Changes in messenger RNA (mRNA) and protein levels in the spinal cords of MIA-treated and saline-treated rats were analyzed using an unpaired *t*-test. Correlations between joint histology and between MIA-induced changes in pain behavior and neuronal responses were performed using a Spearman's 1-tailed rank correlation test.

## RESULTS

### Pain behavior in the MIA model of OA

**Figure 1:**
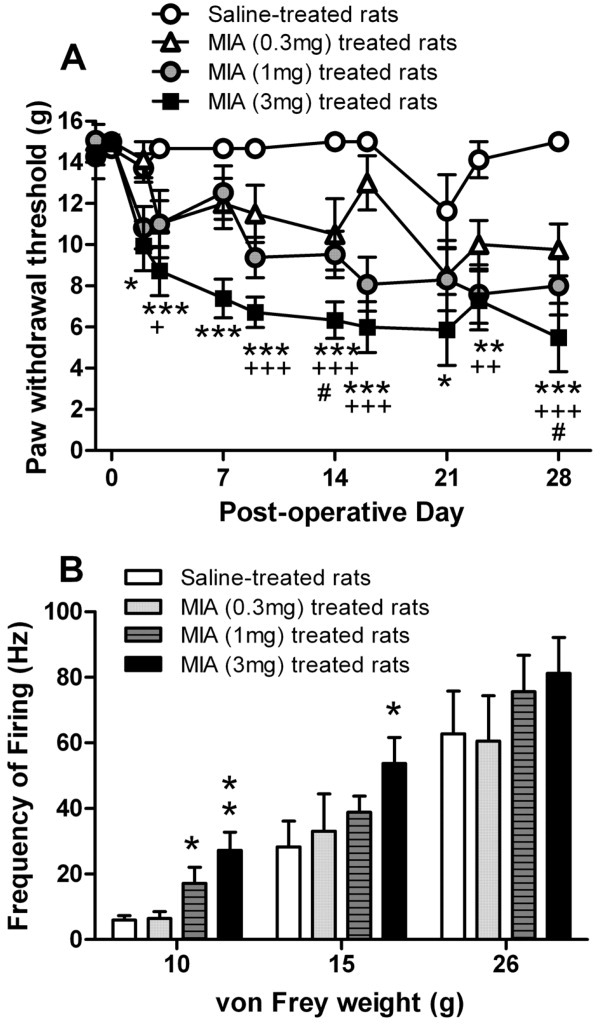
**A,** Changes in hind paw withdrawal threshold to mechanical punctuate stimulation in rats treated with mono-iodoacetate (MIA; 0.3–3 mg) and rats treated with saline. ∗ = *P* < 0.05, ∗∗ = *P* < 0.01, ∗∗∗ = *P* < 0.001, MIA 3 mg versus saline; + = *P* < 0.05, ++ = *P* < 0.01, +++ = *P* < 0.001, MIA 1 mg versus saline; # = *P* < 0.05, MIA 0.3 mg versus saline, by one-way analysis of variance with Bonferroni's post hoc test. **B,** Responses of wide dynamic range neurons to mechanical (10–26 gm) punctate stimulation of the peripheral receptive field on the hind paws of MIA-treated (0.3–3 mg/50 μl) and saline-treated rats on days 28–31. Values are the mean ± SEM. ∗ = *P* < 0.05; ∗∗ = *P* < 0.01 versus saline, by Mann-Whitney test.

Intraarticular injection of 0.3 mg of MIA did not alter weight-bearing, compared with saline treatment, on either day 14 (data not shown) or day 28 (mean ± SEM 82 ± 4% of weight on contralateral hind limb), despite changes in joint pathology in MIA-treated rats on day 28 (Table 1; additional information available from corresponding author). MIA at doses of 1 mg and 3 mg produced changes in joint pathology (Table 1; additional information available from corresponding author) and significantly decreased weight-bearing on the ipsilateral hind limb (*P* < 0.01 for 1 mg–treated rats on days 14 and 28, 80 ± 4% and 77 ± 6% of weight on contralateral hind limb, respectively; *P* < 0.001 for 3 mg–treated rats on days 14 and 28, 67 ± 6% and 66 ± 8% of weight on contralateral hind limb, respectively), compared with saline-treated rats (mean ± SEM 89 ± 9% and 99 ± 3% of weight on contralateral hind limb, respectively). Following intraarticular injection of MIA, hind paw withdrawal thresholds to mechanical punctuate stimulation were significantly decreased compared with those in rats receiving saline treatment, over the course of the study (Figure 1A). Intraarticular injection of MIA did not alter contralateral hind paw withdrawal thresholds (data not shown). Intraarticular injection of saline did not alter weight distribution (data not shown) and hind paw withdrawal thresholds (Figure 1A), nor did it produce any pathologic conditions in the joints (Table 1).

**Table 1:** Severity of changes in the cartilage, subchondral bone, and synovium produced by intraarticular injection of mono-iodoacetate (MIA)*

	Cartilage	Subchondral bone	Synovium
Day 14			
Saline-treated rats (n = 3), median (range) score	0 (0)	0 (0–0.25)	0 (0–0.25)
MIA-treated rats			
1 mg (n = 6), median (range) score	7.75 (0–24.0)	1.42 (0–3.0)	0.75 (0–2.0)
3 mg (n = 4), median (range) score	11.0 (0–24.0)	1.50 (0–3.5)	0.25 (0–2.0)
Weight-bearing, Spearman's r	−0.7188†	−0.7312†	−0.6212‡
Paw withdrawal thresholds, Spearman's r	−0.3545	−0.3068	−0.3754
Day 28			
Saline-treated rats (n = 4), median (range) score	0 (0)	0 (0)	0 (0)
MIA-treated rats			
0.3 mg (n = 8), median (range) score	6.5 (0.75–19)	0.83 (0.25–2.75)	0.5 (0–1.0)
1 mg (n = 8), median (range) score	6.13 (0–24.0)	1.0 (0–3.5)	0 (0–2.0)
3 mg (n = 3), median (range) score	24.0 (6.0–24.0)	3.75 (1.0–3.75)	2.75 (0–3.0)
Weight-bearing, Spearman's r	−0.7491§	−0.7453§	−0.5372†
Paw withdrawal thresholds, Spearman's r	−0.5499†	−0.5883†	−0.3761‡

* Intraarticular injection of 1 mg MIA produced marked and comparable changes in cartilage and subchondral bone on days 14 and 28. The severity of the changes in cartilage, subchondral bone, and synovium produced by intraarticular injection of 3 mg MIA increased from day 14 to day 28. Synovitis was mild following the 1-mg dose on day 28 but was evident following the 3-mg dose on day 28. Note that because 0.3 mg MIA produced no change in pain behavior on day 14, joints were not collected at this time point, and data are not available. MIA-induced changes in weight-bearing were correlated with changes in cartilage, subchondral bone, and synovium histology on both day 14 (n = 13 rats) and day 28 (n = 23 rats) postinjection. Decreases in hind paw withdrawal thresholds were significantly correlated with changes in cartilage subchondral bone and synovium histology on day 28 but not day 14 following injection of MIA.

† *P* < 0.01.

‡ *P* < 0.05.

§ *P* < 0.001.

### Increased excitability of spinal neurons in MIA-treated rats

On the basis of the behavioral data, the effects of 1 mg (7 neurons) and 3 mg (11 neurons) of MIA on neuronal responses were studied on days 14–17. MIA-treated rats exhibited changes in weight-bearing and hind paw withdrawal thresholds prior to the electrophysiologic studies. At this time point, the responses of WDR neurons to mechanical punctuate stimulation of the hind paw were comparable in MIA-treated and saline-treated rats (11 neurons; data not shown). At the later time point (days 28–31), when mechanical allodynia was maximal, the effects of MIA (0.3 mg [8 neurons], 1 mg [9 neurons], and 3 mg [11 neurons]) on the hind paw–evoked responses of WDR neurons were compared with responses in saline-treated rats (9 neurons). The responses of WDR neurons to innocuous and noxious mechanical stimulation (10 gm and 15 gm, respectively) of the hind paw were significantly increased in MIA (1 mg and 3 mg)–treated rats compared with the neuronal responses in saline-treated rats (Figure 1B). Higher-weight (26 gm–evoked) responses of WDR neurons were comparable in MIA-treated rats and saline-treated rats.

**Figure 2:**
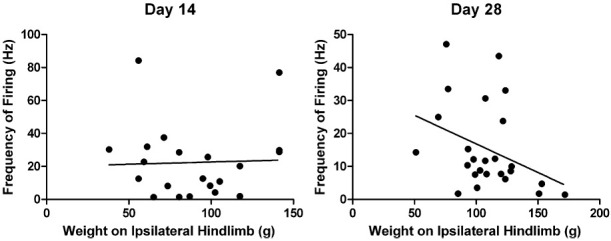
Changes in weight-bearing and hind paw–evoked responses of wide dynamic range (WDR) neurons on days 14 and 28 following mono-iodoacetate (MIA) injection. No correlation between changes in weight-bearing on the MIA-treated joint and innocuous (10 gm) mechanically evoked responses of WDR neurons was observed 14 days following intraarticular injection of MIA (r = −0.0781). At the 28-day time point, there was a significant (*P* < 0.05) correlation between 10 gm–evoked responses of WDR neurons of MIA-treated rats and changes in weight-bearing in these rats (r = −0.4139).

The relationship between joint histology, hyperalgesia (weight-bearing), and allodynia and the responses of WDR neurons was investigated. Changes in cartilage, subchondral bone, and synovium histology were significantly correlated, on both day 14 and day 28 after MIA injection (data not shown). MIA-induced changes in cartilage, subchondral bone, and synovium were significantly correlated with changes in weight-bearing on day 14 and day 28 after MIA injection (Table 1). Interestingly, MIA-induced changes in cartilage, subchondral bone, and synovium were significantly correlated with mechanical allodynia on day 28, but not on day 14, after MIA injection (Table 1). Changes in weight-bearing and hind paw–evoked responses of WDR neurons were significantly correlated on day 28, but not day 14, after MIA injection (Figure 2). These data emphasize the importance of the later time point for studying the mechanisms underlying joint pain in this model.

### Increased spinal cord levels of endocannabinoids in MIA-treated rats

**Figure 3:**
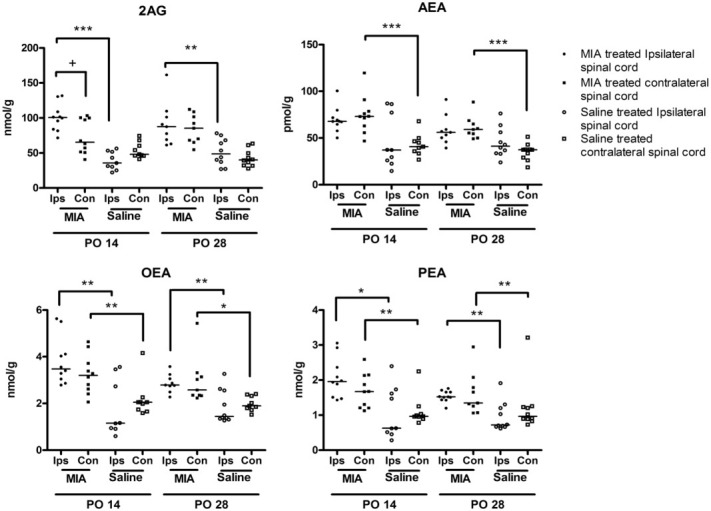
Levels of endocannabinoids (2-arachidonyl glycerol [2-AG] and anandamide [AEA]) and related *N*-acylethanolamines (oleoylethanolamide [OEA] and palmitoylethanolamide [PEA]) in the spinal cords of rats treated with mono-iodoacetate (MIA; 1 mg) or saline, 14 and 28 days following intraarticular injection of MIA. Bars show the median. + and ∗ = *P* < 0.05; ∗∗ = *P* < 0.01; ∗∗∗ = *P* < 0.001, by Mann-Whitney test. Ips = ipsilateral; Con = contralateral; PO = postoperative day.

We investigated whether spinal cord levels of the endocannabinoids are altered in rats treated with 1 mg of MIA. Levels of 2-AG were significantly elevated in the ipsilateral spinal cords of MIA-treated rats compared with saline-treated rats on day 14. Similarly, levels of 2-AG were significantly increased in the ipsilateral spinal cords of MIA-treated rats compared with saline-treated rats on day 28 (Figure 3). A tendency toward bilateral increases in anandamide levels in the spinal cords of MIA-treated rats compared with saline-treated rats was observed at both time points, but significance was reached only for the contralateral spinal cords (Figure 3). Spinal cord levels of the related *N*-acylethanolamines, PEA and OEA, were significantly elevated in the ipsilateral and contralateral spinal cords of MIA-treated rats compared with saline-treated rats, at both time points (Figure 3).

**Figure 4:**
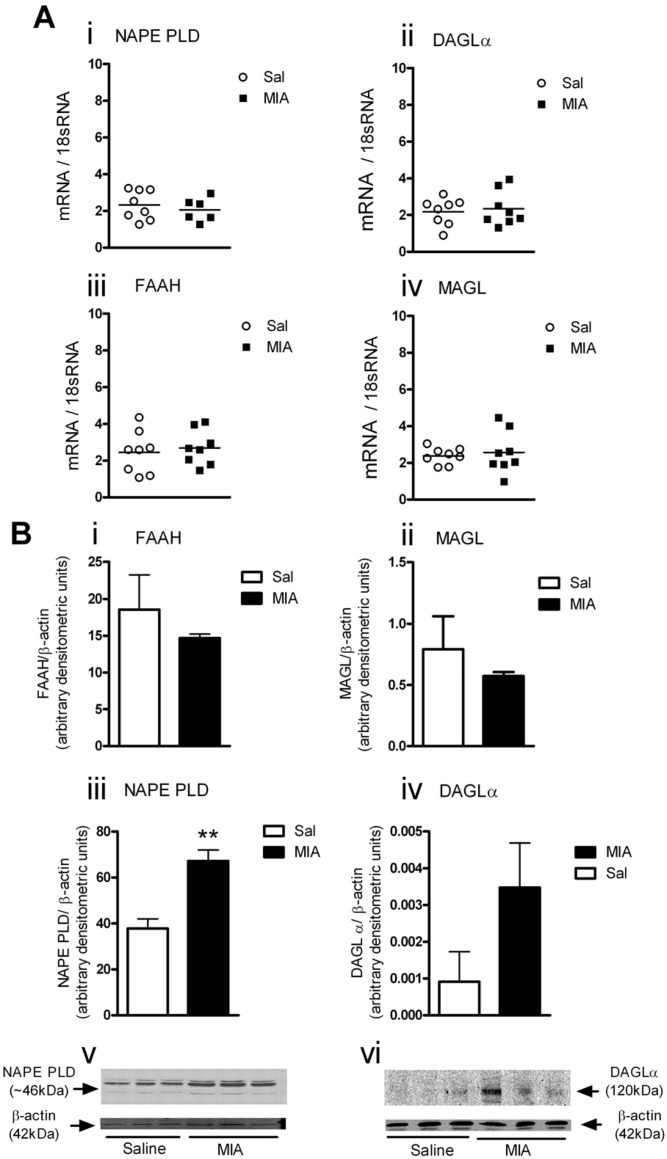
Comparative analysis of gene and protein expression of endocannabinoid-metabolizing enzymes in the ipsilateral spinal cord 28 days following intraarticular injection of mono-iodoacetate (MIA) or saline (Sal). **A,** Messenger RNA expression of *N*-acyl phosphatidylethanolamine phospholipase D (NAPE-PLD), diacylglycerol lipase α (DAGLα), fatty acid amide hydrolase (FAAH), and monoacylglyceride lipase (MAGL) relative to 18S RNA in saline-treated and MIA-treated rats. Bars show the mean. **B, Top,** Densitometric analysis of protein expression for FAAH, MAGL, NAPE-PLD, and DAGLα relative to β-actin in saline-treated and MIA-treated rats. Bars show the mean and SEM. **Bottom,** Immunoblots demonstrating elevated levels of NAPE-PLD and DAGLα, respectively, in MIA-treated rats. NAPE-PLD protein levels were significantly increased in the ipsilateral spinal cord of MIA-treated rats compared with saline-treated rats. There was significant protein expression of DAGLα in the ipsilateral spinal cord of MIA-treated rats, but the expression of DAGLα protein was at or below the limits of detection in saline-treated rats. n = 6–8 rats per treatment group for gene expression data, and n = 3 rats per treatment group for protein data. ∗∗ = *P* < 0.01 versus saline-treated rats, by unpaired *t*-test.

Changes in the levels of endocannabinoids and the related *N*-acylethanolamines are unlikely to be attributable to altered catabolism, because neither FAAH nor MAGL activity was altered in the spinal cords of MIA-treated rats on day 14 (data not shown) or day 28 (for FAAH activity, mean ± SEM 48 ± 7 pmoles/minute/mg protein for the ipsilateral spinal cord and 41 ± 3 pmoles/minute/mg protein for the contralateral spinal cord; for MAGL activity, mean ± SEM 24 ± 3 pmoles/minute/mg protein for the ipsilateral spinal cord and 26 ± 3 pmoles/minute/mg protein for the contralateral spinal cord), compared with saline-treated rats (for FAAH activity, mean ± SEM 50 ± 4 pmoles/minute/mg protein for the ipsilateral spinal cord and 47 ± 2 pmoles/minute/mg protein for the contralateral spinal cord; for MAGL activity, mean ± SEM 31 ± 4 pmoles/minute/mg protein for the ipsilateral spinal cord and 31 ± 7 pmoles/minute/mg protein for the contralateral spinal cord). Furthermore, levels of FAAH and MAGL protein and mRNA in the ipsilateral spinal cords of MIA-treated rats were comparable with levels in saline-treated rats (Figure 4). In contrast, protein levels of NAPE-PLD, the major synthetic enzyme for anandamide, PEA, and OEA, were significantly (mean ± SEM 78 ± 13%) increased in the ipsilateral spinal cords of MIA-treated rats on day 28 compared with saline-treated rats (Figure 4). Levels of NAPE-PLD mRNA were not altered in MIA-treated rats (Figure 4). Consistent with the elevated levels of 2-AG in MIA-treated rats, protein levels of the synthetic enzyme DAGLα on day 28 were higher in the ipsilateral spinal cords of MIA-treated rats compared with saline-treated rats, in which levels of DAGLα were at or below the limits of detection. DAGLα mRNA levels were not altered in MIA-treated rats.

### Spinal endocannabinoids provide greater tonic modulation of neuronal activity in the MIA model of OA pain

**Figure 5:**
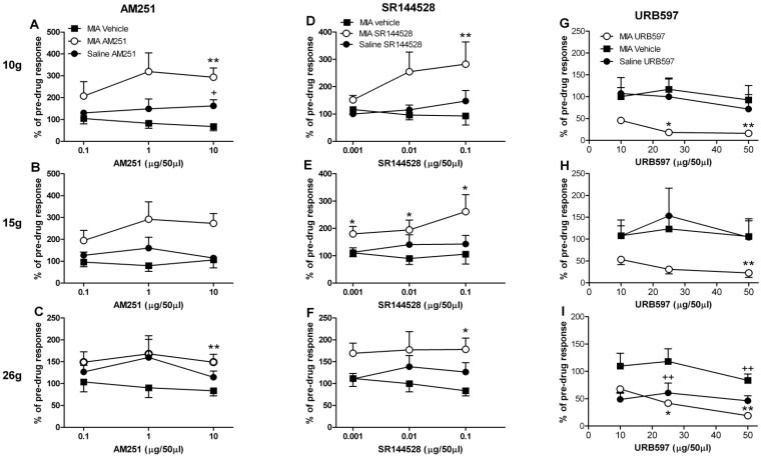
Effects of spinal administration of the spinal cannabinoid 1 (CB_1_) receptor antagonist AM251 (0.1–10 μg/50 μl) (**A**–**C**), the CB_2_ receptor antagonist SR144528 (0.001–0.1 μg/50 μl) (**D**–**F**), and the fatty acid amide hydrolase (FAAH) inhibitor URB597 (10–50 μg/50 μl) (**G**–**I**). Spinal administration of AM251 produced greater facilitation of 10 gm– and 15 gm–evoked responses of spinal neurons in mono-iodoacetate (MIA)–treated rats (n = 6 neurons in 6 rats) compared with saline-treated rats (n = 6 neurons in 6 rats). Spinal administration of SR144528 significantly facilitated mechanically (10–26 gm) evoked responses of wide dynamic range (WDR) neurons in MIA-treated rats (n = 6 neurons in 6 rats) but not saline-treated rats (n = 6 neurons in 6 rats). Spinal administration of URB597 significantly inhibited 10 gm– and 15 gm–evoked responses of WDR neurons in MIA-treated rats (n = 7 neurons in 7 rats) but not saline-treated rats (n = 7 neurons in 7 rats), whereas 26 gm–evoked responses of WDR neurons were inhibited by URB597 in both MIA-treated and saline-treated rats. Data are expressed as mean and SEM maximal percent of the control response. ∗ = *P* < 0.05, ∗∗ = *P* < 0.01 versus vehicle in MIA-treated rats; + = *P* < 0.05, ++ = *P* < 0.01 versus vehicle in saline-treated rats, by Mann-Whitney test.

Because levels of endocannabinoids were elevated in the spinal cords of MIA-treated rats, the potential role of endocannabinoids in tonic modulation of the activity of WDR neurons in MIA-treated rats was investigated. The mechanically evoked responses of WDR neurons prior to administration of vehicle or drug were consistent with those shown in Figure 1B; there were no significant differences between control values for the different treatment groups. Spinal administration of the CB_1_ receptor antagonist AM251 (0.1–10 μg/50 μl) significantly facilitated innocuous (10 gm) and noxious (26 gm) mechanically evoked responses of WDR neurons in MIA-treated rats (Figure 5). Although the highest dose of AM251 (10 μg/50 μl) significantly facilitated 10 gm–evoked responses of dorsal horn neurons in saline-treated rats, the magnitude of the effect of AM251 was 1.8-fold greater in the MIA-treated rats compared with saline-treated rats (Figure 5).

Because CB_2_ receptor expression is up-regulated in the spinal cord in chronic pain states, we also investigated whether these receptors provided tonic control of neuronal responses in the MIA-treated rats. Spinal administration of the CB_2_ receptor antagonist SR144528 significantly facilitated both innocuous (10 gm) and noxious (15–26 gm) mechanically evoked responses of WDR neurons in MIA-treated rats but did not significantly alter evoked responses of WDR neurons in saline-treated rats (Figure 5).

Based on the observation that endocannabinoid levels are elevated but FAAH activity is similar in MIA-treated rats versus saline-treated rats, the final series of experiments investigated whether pharmacologic blockade of the catabolism of anandamide by FAAH, using URB597, had a differential effect in MIA-treated rats compared with saline-treated rats. Administration of URB597 (25–50 μg/50 μl) onto the spinal cord significantly inhibited mechanically evoked responses of WDR neurons in MIA-treated rats (Figure 5). The inhibitory effects of URB597 on 10 gm–evoked responses were significantly (*P* < 0.05) greater in MIA-treated rats compared with saline-treated rats. URB597 had a comparable inhibitory effect on the noxious (26 gm) mechanically evoked responses of WDR neurons in MIA-treated rats and saline-treated rats.

## DISCUSSION

This study is the first to assess the temporal effects of MIA-induced knee joint pathology on pain behavior and the responses of spinal dorsal horn neurons. Although behavioral pain responses occurred from day 7 onward, it was only between 28 days and 31 days following MIA treatment that spinal neuronal responses were facilitated, suggestive of spinal hyperexcitability. The significant correlation between MIA-induced changes in pain behavior (weight-bearing) and low weight–evoked responses of neurons 28 days following MIA injection provides evidence for an association between pain behavior and neuronal responses at the later time point. There was a significant correlation between changes in subchondral bone histology and mechanical allodynia on day 28, which supports the proposal that bone pathology contributes to pain responses and central sensitization in OA (25). Collectively, the decreased hind paw withdrawal thresholds and increased responses of spinal neurons innervating sites distal to the site of injury in MIA-treated rats are consistent with the referred pain experienced by patients with OA (26).

A novel finding of this study is that the levels of anandamide, 2-AG, PEA, and OEA were elevated in the spinal cords of MIA-treated rats. Neither mRNA/protein levels nor the activities of FAAH and MAGL (the major catabolic pathways for anandamide and 2-AG) were altered in the spinal cords of MIA-treated rats compared with saline-treated control rats. Protein levels of NAPE-PLD and DAGLα, the major synthetic enzymes for the *N*-acylethanolamines (anandamide, PEA, and OEA) and 2-AG, respectively, were increased in the spinal cords of MIA-treated rats compared with saline-treated rats. Thus, the increased spinal cord levels of anandamide, PEA, OEA, and 2-AG in MIA-treated rats most likely arose as the result of increased synthesis via NAPE-PLD and DAGLα, respectively. The levels of DAGLα protein extracted from the spinal cords of saline-treated rats were observed to be below the level of detection by Western immunoblotting. DAGLα has, however, been shown to be present in postsynaptic dorsal horn neurons of the spinal cord (27).

Increased mechanical responses of afferent fibers (14,28,29) have been described in the MIA model. We propose that this increased sensory input into the spinal cord drives the observed facilitations in spinal cord neuronal responses in MIA-treated rats, resulting in the increased capacity of NAPE-PLD and DAGLα to synthesize anandamide and 2-AG, respectively. The bilateral changes in the levels of *N*-acylethanolamines in MIA-treated rats suggest that additional input into the spinal cord may also contribute. Increased descending facilitation from the rostral ventromedial medulla, which has bilateral influence on spinal cord function in other models of chronic pain (30) and is activated in patients with OA (2), may also contribute.

Interestingly, mRNA levels of NAPE-PLD and DAGLα were not elevated alongside the protein levels. There are limited data regarding the relationship between mRNA and protein expression for NAPE-PLD and DAGLα, although both enzymes can be regulated at the transcription level (31,32). In the MIA model, however, it appears that regulation occurs posttranscriptionally, possibly at the level of translation or protein degradation, and this possibility merits further investigation.

Pharmacologic intervention with a CB_1_ receptor antagonist provided evidence that the elevated spinal cord levels of anandamide and 2-AG have a functional role in this model of OA pain. Low weight (10 gm)–evoked responses of spinal neurons in the MIA-treated rats were facilitated by the CB_1_ receptor antagonist to a greater extent than that seen in saline-treated rats. These data are consistent with the established role of the endocannabinoids in the tonic modulation of spinal neuronal activity (33) and indicate that the elevated spinal cord levels of endocannabinoids have a functional role in limiting increases in the excitability of spinal neurons, in particular those to low-weight mechanical stimuli, which produce allodynia in this model. It is noteworthy that CB_1_ receptor blockade did not modulate spinal cord responses to noxious knee compression in a 14-day model of monarticular inflammation (34), which may reflect differences in progression or the type of model studied.

Remarkably, our functional data indicated that along with CB_1_ receptors, spinal CB_2_ receptors are also activated by elevated endocannabinoid levels in MIA-treated rats but not in saline-treated rats. The ability of a spinally administered CB_2_ receptor antagonist to facilitate evoked neuronal responses in MIA-treated rats provides the first functional evidence for a spinal cord–related role of CB_2_ receptors in this model of OA. CB_2_ receptor expression is increased and is associated with activated microglia in the spinal cord in other models of chronic pain (35,36); furthermore, spinally administered CB_2_ receptor agonists are antinociceptive in models of neuropathic pain (37; for review, see refs.^38^ and^39^). Our data suggest that the potential role(s) of spinal cord CB_2_ receptors in models of OA pain are worthy of further investigation, and that this receptor may be a novel target for the inhibition of OA-related pain.

Inhibitors of the enzymes responsible for catabolism of the endocannabinoids have therapeutic potential as novel analgesic treatments (16; for review, see ref.^40^), the rationale being that chronic pain states are associated with discrete increases in the levels of endocannabinoids (for review, see ref.^10^), and that preventing their catabolism could provide analgesic effects, with limited CB_1_ receptor–mediated side effects. Spinal administration of the FAAH inhibitor URB597 produced dose-related inhibition of the evoked responses of spinal neurons in MIA-treated rats. The inhibitory effects of URB597 on low weight–evoked responses of spinal neurons were greater in MIA-treated rats compared with saline-treated rats, which is consistent with the increased spinal cord levels of anandamide in MIA-treated rats and with CB_1_ and CB_2_ receptor antagonists having greater effects on low weight–evoked responses of spinal neurons in MIA-treated rats versus saline-treated rats. These data suggest that the elevated spinal cord levels of endocannabinoids play an important role in the tonic modulation of low-weight (allodynic) input in this model of OA pain, which can be potentiated by reducing their catabolism by FAAH. Our group previously demonstrated, using identical experimental protocols in neuropathic rats, that spinal administration of the highest dose of URB597 used in this study elevated levels of anandamide in the spinal cord, and that the inhibitory effects of this dose of URB597 on neuronal responses were blocked by a CB_1_ receptor antagonist (16).

In conclusion, we have shown that the joint pathology and pain behavior produced by intraarticular injection of MIA is associated with increased excitability of spinal neurons. This experimental model of OA pain was associated with increased levels of endocannabinoids and NAPE-PLD and DAGLα protein, both of which contribute to the synthesis of these endocannabinoids. The elevated spinal cord levels of endocannabinoids appeared to have a greater functional inhibitory role in the modulation of neuronal activity via CB_1_ and CB_2_ receptors, in particular for low-weight mechanical inputs, in the MIA model of OA. Consistent with these data, spinal inhibition of FAAH had significantly greater effects on low weight–evoked responses of spinal neurons in MIA-treated rats. These mechanistic studies have clinical implications, especially in the context of mechanical allodynia, which is a major symptom of chronic pain states and is poorly responsive to treatment with conventional analgesics. Our study provides new evidence for a crucial role of the endocannabinoids in controlling neuronal excitability at the level of the spinal cord in a clinically relevant model of OA pain.
